# Development and validation of a risk prediction model for major postoperative adverse events in elderly colon cancer using firth regression: a large-scale retrospective study

**DOI:** 10.3389/fonc.2026.1870296

**Published:** 2026-07-23

**Authors:** Yixiang Huang, Xiaohui Yuan, Xiangbo Zhang, Runda Jiao, Cheng Li, Jiacheng Xu, Tianyi Zhang, Lihua Liu

**Affiliations:** 1 Graduate School, Chinese PLA General Hospital, Beijing, China; 2 Department of Medical Innovation and Research, Chinese PLA General Hospital, Beijing, China

**Keywords:** adverse events, colon cancer, elderly, firth regression, medical record homepage

## Abstract

**Background:**

Elderly patients with colon cancer are at increased risk of major postoperative adverse events, but existing risk assessment tools often require detailed clinical or laboratory information. We developed and internally validated a rapid prediction nomogram using routinely available administrative variables.

**Methods:**

This multicenter retrospective study included 4, 942 elderly patients with colon cancer from five hospitals. Major postoperative adverse events were identified using a predefined ICD-10 code-based algorithm adapted from the Healthgrades patient-safety framework. Given the low event rate, Firth penalized likelihood regression was used to develop the prediction model. Internal validation was performed using 1, 000 bootstrap resamples. Model performance was assessed by discrimination, calibration, Brier score, and decision curve analysis.

**Results:**

Independent predictors included payment method (medical insurance: OR = 2.630), admission type (outpatient: OR = 0.501), surgical approach (minimally invasive: OR = 0.473), and comorbidity index (OR = 1.041). The full model showed moderate discrimination, with an apparent AUC of 0.724 and a bootstrap-corrected AUC of 0.709. The apparent and bootstrap-corrected Brier scores were 0.0244 and 0.0247, respectively. Decision curve analysis suggested potential net benefit across threshold probabilities of 1%–51%.

**Conclusion:**

This nomogram may serve as a rapid, low-cost preliminary screening tool for estimating the risk of major postoperative adverse events in elderly patients with colon cancer. External validation and further refinement using more detailed clinical, nutritional, oncological, and functional variables are needed before broader clinical application.

## Introduction

According to the latest data from the International Agency for Research on Cancer (IARC) in 2022, there were over 1.9 million new cases of colon cancer and 930, 000 deaths globally in 2020, making it the third most commonly diagnosed cancer and the second leading cause of cancer-related death worldwide (1). By 2040, the global burden of colon cancer is projected to rise to 3.2 million new cases and 1.6 million deaths, with the majority of cases occurring in countries with a high Human Development Index (2). The disease burden of colon cancer in China is equally severe; in 2022, there were approximately 517, 000 new cases and 240, 000 deaths, ranking second in incidence and fourth in mortality among all malignant tumors in China (3). Furthermore, colon cancer is considered to have the second-highest treatment costs, accounting for 12.6% of all cancer treatment expenditures (4). Studies have shown that patients who experience postoperative adverse events following colon cancer surgery face a range of physical, social, sexual, and psychological challenges (5).

Currently, with the deepening of population aging in China, the burden of multimorbidity is becoming increasingly critical. Improving clinical outcomes for elderly patients with multimorbidity has become an urgent priority for healthcare professionals. However, existing studies are predominantly based on single-center data and primarily focus on the predictive value of laboratory and imaging indicators for postoperative adverse events. There is a paucity of research utilizing administrative health data for rapid risk assessment. This study utilizes large-sample clinical data to investigate the risk factors associated with adverse events during hospitalization. We aim to construct and validate a risk prediction model for adverse events in hospitalized colon cancer patients, providing a quantitative tool for the rapid identification of high-risk populations and offering evidence-based support for personalized prevention and intervention strategies.

## Materials and methods

### Study design and participants

This retrospective cohort study was conducted using administrative health data retrieved from the Hospital Information Systems (HIS) of five tertiary Grade-A hospitals in Beijing between January 2014 and December 2023. The baseline demographic and clinical characteristics stratified by participating center are provided in **Supplementary Table 1**. The study enrolled elderly patients (aged ≥60 years) with a primary diagnosis of colon cancer, identified by the International Statistical Classification of Diseases and Related Health Problems, 10th Revision (ICD-10) code C18. Inclusion criteria further required patients to have complete medical record data; for patients with multiple readmissions, only the first hospitalization for colon cancer was recorded as the index admission. Patients with missing clinical data were excluded. A total of 4, 942 eligible patients were ultimately included and stratified into an adverse events group (n=129) and a non-adverse events group (n=4, 813) based on the occurrence of postoperative adverse events. Due to the nature of de-identified data, ethics approval and informed consent from participants were exempt from institutional review board. The study was conducted and reported in compliance with the Strengthening the Reporting of Observational Studies in Epidemiology (STROBE) guidelines.

### Variables

The primary outcome was defined as the occurrence of postoperative adverse events during the index hospitalization. Postoperative adverse events included acute respiratory failure, acute myocardial infarction, acute renal injury, acute venous embolism, acute infection, sepsis, and in-hospital death. These adverse events were identified using an ICD-10 code-based algorithm adapted according to the Healthgrades patient-safety framework (6). The detailed ICD-10 codes used to identify major postoperative adverse events are provided in **Supplementary Table 2**.

To further assess the robustness of the prediction model, in-hospital unplanned return to the operating room was used as a secondary outcome. This endpoint is consistent with surgical quality and safety monitoring practice and was defined as an unplanned repeat operation required during the index hospitalization after the index operation because of a postoperative complication or an untoward outcome related to the initial surgery (7). As an objectively documented and clinically significant endpoint, unplanned return to the operating room has been widely used as an indicator of surgical quality and patient safety.

The covariates included gender, age, admission type, comorbidity index, tumor location, payment method (insurance status), marital status, and surgical approach. Additionally, the Comorbidity Index was utilized to quantify the burden of comorbidities (8).

### Statistical analysis

Data processing was performed using R 4.4.3. Continuous variables were expressed as median (interquartile range) and compared between groups using the Mann-Whitney U test. Categorical variables were presented as frequency [n (%)] and analyzed using the Chi-square χ² test or Fisher’s exact test.

To identify independent predictors of postoperative adverse events, multivariate analysis was conducted using Firth penalized likelihood regression, which is recommended for reducing small-sample bias and improving the stability of coefficient estimation in settings with sparse outcome events (9). A two-sided *P*-value < 0.05 was considered statistically significant. Given the relatively low event rate (2.61%), randomly splitting the dataset into training and validation cohorts was not employed, as it would severely reduce statistical power. Instead, a rigorous internal validation was performed using the bootstrap resampling method with 1, 000 iterations. To avoid predictor selection bias, a prognostic nomogram was constructed based on the full multivariate Firth regression model encompassing all prespecified clinical variables (10). Model discrimination was assessed using the receiver operating characteristic (ROC) curve, with both apparent and bootstrap-corrected areas under the curve (AUCs) reported. Overall prediction accuracy was evaluated using the Brier score, including both apparent and bootstrap-corrected estimates. Calibration was evaluated using a bootstrap-resampled calibration plot. To improve bedside applicability, a simplified point-score chart and a total-points-to-risk conversion curve were derived from the final prediction model and provided in the **Supplementary Figure 1**. Decision curve analysis (DCA) was performed using the ‘rmda’ package to evaluate clinical net benefit, and secondary outcome analysis using unplanned return to the operating room was performed to assess the robustness of the model findings.

## Results

### Patient characteristics

A total of 4, 942 elderly patients with colon cancer were included in this study. Among them, 129 patients (2.61%) experienced postoperative adverse events (the adverse events group), while the remaining 4, 813 patients did not (the non-adverse events group).

There was no statistically significant difference in gender between the two groups (*P* > 0.05). However, significant differences were observed in age, admission type, comorbidity index, tumor location, payment method, surgical approach, and marital status (all *P* < 0.05). The baseline clinical characteristics of the study population are summarized in **Table 1**.

**Table 1:** Clinical characteristics of elderly patients with colon cancer.

Variable	Total (n=4942)	Adverse events group (n=129)	Non-adverse events group (n=4813)	*P-*value
Age (years)	69.00 (64.00, 74.00)	71.00 (65.00, 77.00)	69.00 (64.00, 74.00)	0.012
Gender				0.244
Male	2973 (60.16%)	84 (65.12%)	2889 (60.02%)	
Female	1969 (39.84%)	45 (34.88%)	1924 (39.98%)	
Admission type				<.001
Outpatient	4255 (86.10%)	91 (70.54%)	4164 (86.52%)	
Emergency	687 (13.90%)	38 (29.46%)	649 (13.48%)	
Comorbidity Index	0.00 (0.00, 14.00)	9.00 (0.00, 21.00)	0.00 (0.00, 14.00)	<.001
Tumor location				0.002
Left-sided colon	2415 (48.87%)	62 (48.06%)	2353 (48.89%)	
Right-sided colon	2151 (43.52%)	47 (36.43%)	2104 (43.71%)	
Unspecified	376 (7.61%)	20 (15.50%)	356 (7.40%)	
Payment method				<.001
Self-pay	1712 (34.64%)	20 (15.50%)	1692 (35.15%)	
Medical Insurance	3230 (65.36%)	109 (84.50%)	3121 (64.85%)	
Surgical approach				<.001
Minimally Invasive	3665 (74.16%)	72 (55.81%)	3593 (74.65%)	
Open	1277 (25.84%)	57 (44.19%)	1220 (25.35%)	
Marital status				0.019
Married	4837 (97.88%)	122 (94.57%)	4715 (97.96%)	
Single/Divorced/Widowed	105 (2.12%)	7 (5.43%)	98 (2.04%)	

### Screening for predictive factors

Univariate Firth regression analysis was performed to identify potential predictors of postoperative adverse events in elderly patients with colon cancer. The results indicated that age (95% CI: 1.014–1.062, *P* = 0.002), admission type (95% CI: 0.252–0.545, *P* < 0.001), payment method (95% CI: 1.800–4.659, *P* < 0.001), surgical approach (95% CI: 0.301–0.609, *P* < 0.001), comorbidity index (95% CI: 1.036–1.065, *P* < 0.001), unspecified tumor location (95% CI: 1.299–3.610, *P* = 0.005), and marital status (95% CI: 1.366–6.291, *P* = 0.016) were associated with the risk of adverse events (**Table 2**).

**Table 2:** Univariate and multivariate firth regression analysis of risk factors for postoperative adverse events in elderly colon cancer patients.

	Univariate analysis		Multivariate analysis	
Variable	OR (95%*CI*)	*P*-value	OR (95%*CI*)	*P*-value
Gender				
Male	Ref		Ref	
Female	0.808 (0.561-1.165)	0.249	0.705 (0.478-1.028)	0.070
Age	1.038 (1.014-1.062)	0.002	1.019 (0.994-1.043)	0.139
Admission type				
Emergency	Ref		Ref	
Outpatient	0.370 (0.252-0.545)	<.001	0.501 (0.335-0.765)	0.002
Payment method				
Self-pay	Ref		Ref	
Medical Insurance	2.896 (1.800-4.659)	<.001	2.630 (1.649-4.397)	<.001
Surgical approach				
Open	Ref		Ref	
Minimally Invasive	0.428 (0.301-0.609)	<.001	0.473 (0.324-0.692)	<.001
Comorbidity Index	1.050 (1.036-1.065)	<.001	1.041 (1.026-1.056)	<.001
Tumor location				
Left-sided colon	Ref		Ref	
Right-sided colon	0.850 (0.580-1.245)	0.402	0.777 (0.522-1.149)	0.206
Unspecified	2.165 (1.299-3.610)	0.005	1.402 (0.805-2.348)	0.224
Marital status				
Married	Ref		Ref	
Single/Divorced/Widowed	2.931 (1.366-6.291)	0.016	1.901 (0.769-4.108)	0.153

Subsequently, a multivariate Firth regression analysis was conducted, incorporating age, gender, admission type, comorbidity index, tumor location, payment method, surgical approach, and marital status as independent variables, with the occurrence of adverse events as the dependent variable (0=no adverse event, 1=adverse event). The multivariate analysis revealed that admission type (95% CI: 0.335–0.765, *P* = 0.002), payment method (95% CI: 1.649–4.397, *P* < 0.001), surgical approach (95% CI: 0.324–0.692, *P* < 0.001), and comorbidity index (95% CI: 1.026–1.056, *P* < 0.001) were independent predictors of postoperative adverse events (**Table 2**).

### Construction and validation of the prediction model

**Figure 1:**
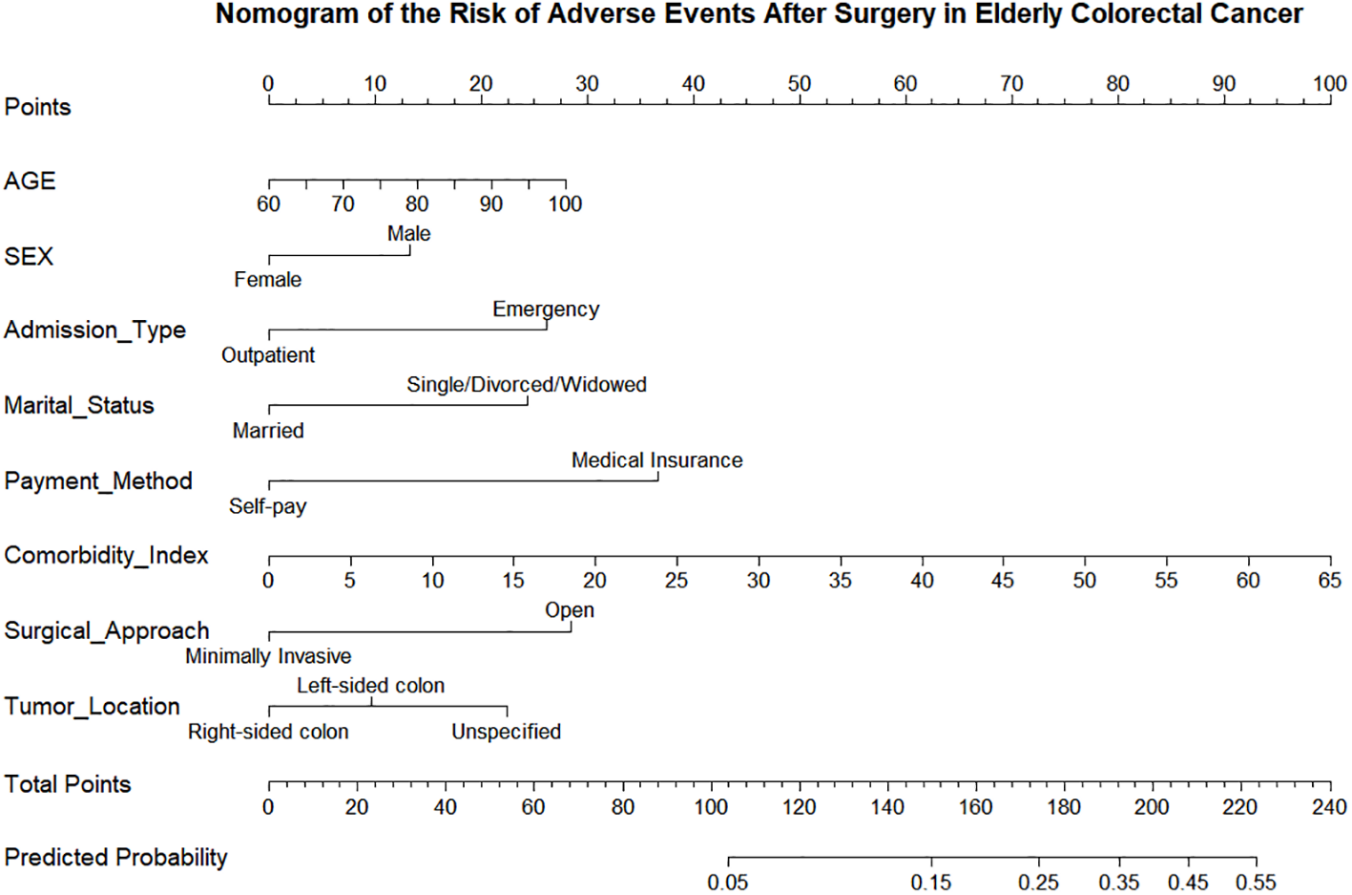
Nomogram for predicting the probability of postoperative adverse events in elderly patients with colon cancer.

Based on the full multivariate Firth regression model encompassing all prespecified clinical predictors, a prognostic nomogram was constructed to predict the risk of postoperative adverse events in elderly patients with colon cancer (**Figure 1**). In this nomogram, the length of the horizontal line corresponding to each predictor visually reflects its relative weight (contribution) to the overall risk of postoperative adverse events. To facilitate bedside application, a simplified point-score chart and total-points-to-risk conversion curve derived from the full multivariable Firth regression model are provided in **Supplementary Figure 1**.

**Figure 2:**
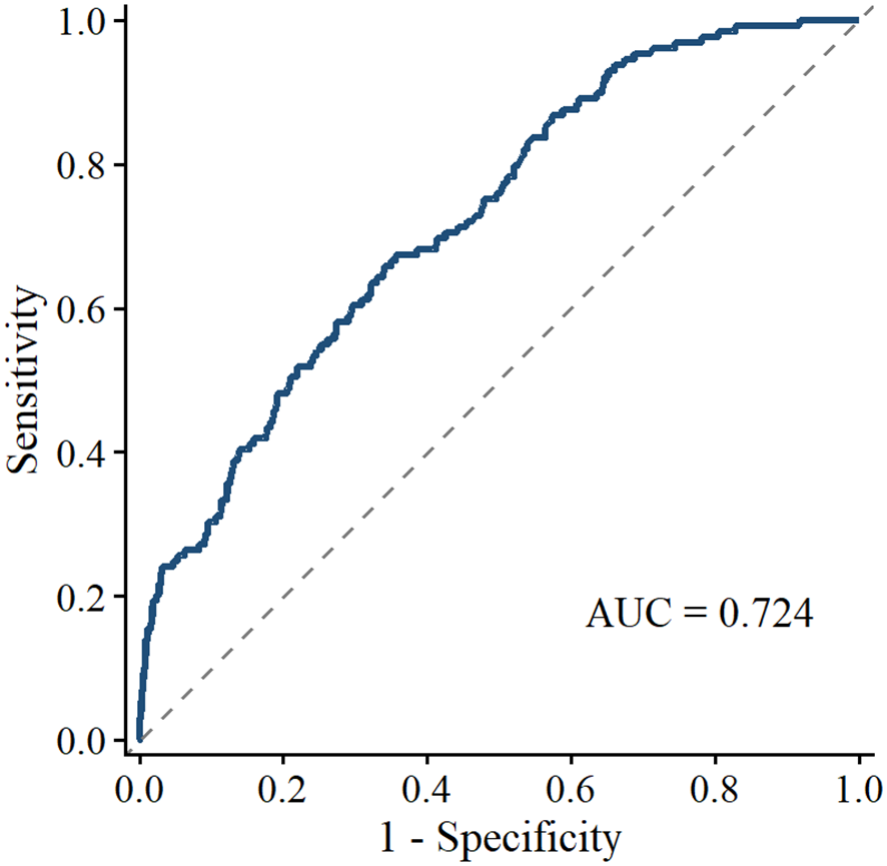
Receiver operating characteristic (ROC) curve of the prediction model for postoperative adverse events in elderly patients with colon cancer.

**Figure 3:**
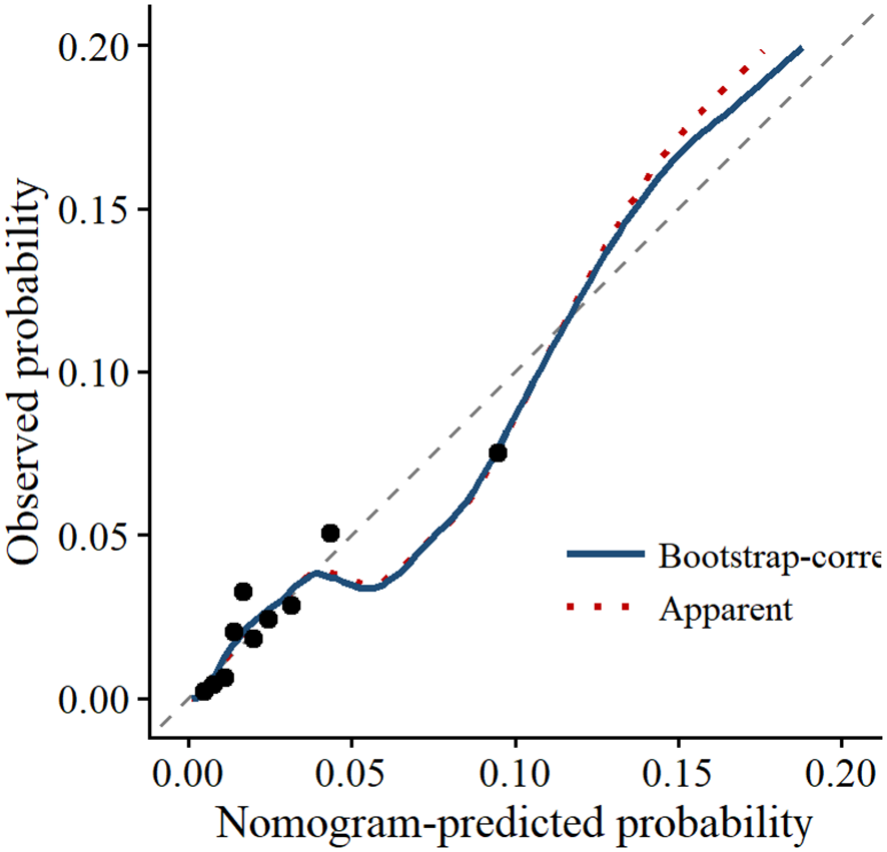
Bootstrap-resampled calibration curve of the prediction model.

The multivariable Firth regression model yielded an apparent area under the receiver operating characteristic curve (AUC) of 0.724, indicating moderate discrimination for identifying elderly hospitalized colon cancer patients at increased risk of major postoperative adverse events (**Figure 2**). Internal validation was performed using 1, 000 bootstrap resamples to estimate optimism-corrected model performance. After bootstrap correction, the AUC was 0.709. To further assess overall prediction accuracy, the Brier score was calculated; the apparent and bootstrap-corrected Brier scores were 0.0244 and 0.0247, respectively. These findings indicate moderate discriminative ability and low overall prediction error. The bootstrap-resampled calibration curve showed reasonable agreement between the nomogram-predicted probabilities and the observed risk of postoperative adverse events, particularly within the lower predicted-risk range (**Figure 3**).

### Decision curve analysis

**Figure 4:**
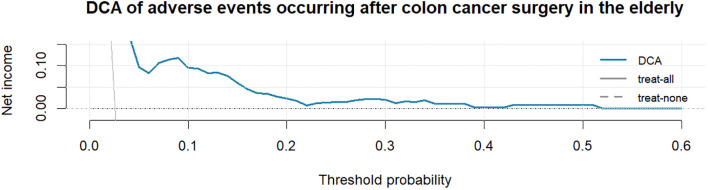
Decision curve analysis (DCA) of the prediction model for postoperative adverse events in elderly patients with colon cancer.

Decision curve analysis indicated that the model provided a positive net benefit across threshold probabilities of 1%–51% compared with the “treat-all” and “treat-none” strategies (**Figure 4**). This suggests potential clinical utility for risk-stratified monitoring, although prospective validation is needed before clinical implementation.

### Secondary outcome analysis

In comparison with the primary model, using “unplanned return to the operating room” as a secondary outcome, the analysis revealed that gender (95% CI: 0.367–0.985, *P* = 0.043), payment method (95% CI: 1.668–5.795, *P* < 0.001), and surgical approach (95% CI: 0.334–0.879, *P* = 0.014) were independent predictors of the secondary outcome (**Table 3**).

**Table 3:** Secondary outcome analysis: risk factors for in-hospital unplanned return to the operating room.

Variable	OR (95% CI)	P-value
Gender		
Male	Ref	
Female	0.611 (0.367–0.985)	0.043
Age	0.982 (0.950–1.014)	0.276
Admission type		
Emergency	Ref	
Outpatient	0.714 (0.415–1.297)	0.258
Payment method		
Self-pay	Ref	
Medical Insurance	2.986 (1.668–5.795)	< 0.001
Surgical approach		
Open	Ref	
Minimally Invasive	0.537 (0.334–0.879)	0.014
Comorbidity Index	1.019 (0.998–1.039)	0.073
Tumor location		
Left-sided colon	Ref	
Right-sided colon	0.681 (0.411–1.106)	0.121
Unspecified	1.223 (0.595–2.328)	0.566
Marital status		
Married	Ref	
Single/Divorced/Widowed	1.433 (0.288–4.434)	0.608

## Discussion

Using a large multicenter retrospective cohort, this study developed and internally validated a risk prediction model for major postoperative adverse events in elderly patients with colon cancer. The model incorporates sex, age, admission type, comorbidity index, tumor location, payment method, marital status, and surgical approach. Given its moderate discrimination and low-cost data requirements, the model is best interpreted as a preliminary screening tool to support early risk stratification rather than a definitive tool for individual risk prediction. The use of Firth penalized likelihood regression was appropriate for this low-event-rate setting. Unlike conventional logistic regression, which may yield biased or unstable estimates when outcome events are sparse, Firth regression applies a penalized likelihood approach to reduce small-sample bias and improve coefficient stability.

The predictors retained in the model should be interpreted primarily from a risk-stratification perspective rather than as causal factors. Emergency admission, open surgery, and a higher comorbidity index may reflect greater acute illness, operative complexity, and multimorbidity burden, respectively. Payment method showed the largest odds ratio, but this variable should be interpreted cautiously and not as a direct causal determinant of postoperative adverse events. It may serve as a surrogate marker for unmeasured factors, including referral pathways, healthcare access, reimbursement policies, nutritional status, socioeconomic characteristics, and disease severity (11).

Although advanced age was associated with postoperative adverse events in the univariate analysis, this association was attenuated after adjustment for other clinical variables. This finding suggests that chronological age alone may be less informative than overall physiological vulnerability and comorbidity burden in elderly patients with colon cancer. Multimorbidity, defined as the coexistence of two or more chronic conditions in the same patient, is common in older adults and may contribute substantially to perioperative risk (12). Consistently, expert consensus has emphasized that treatment decisions for elderly colorectal cancer patients should be guided by comprehensive geriatric assessment rather than strict age thresholds (13). Therefore, perioperative risk stratification in this population should place greater emphasis on comorbidity burden and global functional reserve rather than age alone.

Admission type was another readily available predictor in the model. Compared with emergency admission, outpatient admission was associated with a lower risk of major postoperative adverse events (OR = 0.501, 95% CI: 0.335–0.765, *P* = 0.002). This association is clinically plausible, as emergency presentations of colon cancer are often related to bowel obstruction, perforation, bleeding, or acute deterioration, which may be accompanied by dehydration, electrolyte imbalance, systemic inflammation, and reduced physiological reserve (14). Previous evidence has also shown that emergency presentation is associated with worse surgical outcomes and higher mortality in patients with colorectal cancer (15).

Minimally invasive surgery was associated with a lower risk of major postoperative adverse events compared with open surgery (OR = 0.473, 95% CI: 0.324–0.692, P<0.001). This finding is consistent with previous evidence suggesting that minimally invasive colorectal surgery may reduce surgical trauma and postoperative recovery burden in elderly patients (16, 17). However, this association should be interpreted cautiously because surgical approach is not randomly assigned in retrospective data. Patients selected for minimally invasive surgery may have earlier-stage disease, better baseline condition, fewer adhesions, lower operative complexity, or greater physiological reserve, whereas open surgery may be preferentially used for more complex or urgent cases (18, 19). Therefore, the observed association likely reflects both the potential benefits of minimally invasive techniques and underlying selection by disease severity and patient fitness.

The model demonstrated moderate discrimination after internal validation, with a bootstrap-corrected AUC of 0.709. Given its reliance on routinely available administrative variables, the model is best positioned as a rapid, low-cost preliminary screening tool rather than a definitive instrument for individual risk prediction. It may help clinicians identify elderly colon cancer patients who warrant closer perioperative assessment or subsequent comprehensive geriatric evaluation. To facilitate clinical interpretation, the model was presented as a nomogram, a commonly used tool for individualized risk estimation in clinical prediction research (20, 21). In response to the need for more practical bedside use, we further derived a simplified point-score chart and total-points-to-risk conversion curve, which are provided in **Supplementary Figure 1**.

Decision curve analysis suggested a potential net benefit of the nomogram across threshold probabilities of 1%–51% compared with the “treat-all” and “treat-none” strategies. This finding supports the possible use of the model for risk-stratified postoperative monitoring, while avoiding overinterpretation as direct evidence of improved clinical outcomes (22, 23). Prospective validation is still required before the model can be used to guide clinical decision-making.

The secondary outcome analysis used unplanned return to the operating room as a more specific and objectively documented severe postoperative endpoint, which commonly reflects complications requiring additional operative management, such as anastomotic leakage, postoperative hemorrhage, or wound dehiscence (24). Payment method and surgical approach remained associated with this endpoint, supporting their robustness as predictive signals, although these variables should not be interpreted causally. Payment method may reflect unmeasured healthcare access or referral-related factors, whereas surgical approach may reflect both operative technique and patient selection. Sex was associated with the secondary outcome but not with the primary outcome, a discrepancy that may be related to endpoint specificity and the limited number of events; any biological explanation remains exploratory (25).

This study has several limitations. First, because this was a retrospective study based on routinely collected administrative data, selection bias, information bias, and residual confounding cannot be fully avoided (26). Although the primary outcome focused on major, objectively coded in-hospital adverse events, the use of ICD-10 codes and administrative outcome fields may still introduce potential misclassification or under-ascertainment. Second, some clinically important variables, such as body mass index, serum albumin, tumor stage, functional status, frailty, operative time, blood loss, and detailed perioperative management, were not available in the current dataset. This limitation is particularly relevant to the interpretation of payment method and surgical approach, which may partly reflect unmeasured socioeconomic, clinical, or disease-severity factors rather than direct causal effects. Third, the outcome was based on ICD-coded postoperative adverse events and therefore focused on clinically significant events requiring active medical attention; minor or self-limiting complications may not have been fully captured. Fourth, data were obtained from five tertiary hospitals in Beijing, and the model has not yet been externally validated in independent geographic, temporal, or lower-tier hospital cohorts. Therefore, its generalizability remains uncertain. Finally, this model focused on short-term postoperative adverse events and did not assess long-term oncological outcomes such as overall survival or disease-free survival. Future studies should incorporate richer clinical variables and external validation cohorts to further evaluate the transportability and clinical usefulness of this preliminary screening model.

## Conclusion

In summary, this study developed and internally validated a rapid, low-cost nomogram for estimating the risk of major postoperative adverse events in elderly patients with colon cancer. Based on routinely available administrative variables, the model showed moderate discrimination and may serve as a preliminary screening tool to support early perioperative risk stratification. Further external validation in independent cohorts and incorporation of more detailed clinical, nutritional, oncological, and functional variables are needed before broader clinical application.

## Data Availability

The datasets generated and/or analyzed during the current study are available from the corresponding author upon reasonable request, subject to institutional approval.
