# Supplementary material for: Development and validation of a risk prediction model for major postoperative adverse events in elderly colon cancer using firth regression: a large-scale retrospective study
Source: Front Oncol. 2026 Jul 23;16:1870296. doi: 10.3389/fonc.2026.1870296 (PMC13441950; doi:10.3389/fonc.2026.1870296)
Supplement: Supplementary file 1 [file DataSheet1.docx]

**Supplementary Material**

**Table of Contents**

**Table S1. Baseline demographic and clinical characteristics of the study population stratified by participating center.**

**Table S2. ICD-10 Codes and outcome fields for Identification of Major Postoperative Adverse Events.**

**Figure S1. Point-score chart derived from the prediction model.**

**Table S1 Baseline demographic and clinical characteristics of the study population stratified by participating center**

| Variable | Total (n=4942) | Center A (n=4188) | Center B (n=117) | Center C (n=586) | Center D (n=44) | Center E (n=7) | *P-*value |
| --- | --- | --- | --- | --- | --- | --- | --- |
| Age (years) | 69.00(64.00,74.00) | 69.00(64.00,74.00) | 68.00(64.00,74.00) | 69.50(65.00,75.00) | 70.50(66.00,75.00) | 78.00(68.00,83.50) | 0.008 |
| Gender |  |  |  |  |  |  | 0.258 |
| Male | 2973(60.16) | 2534(60.51) | 73(62.39) | 342(58.36) | 20(45.45) | 4(57.14) |  |
| Female | 1969(39.84) | 1654(39.49) | 44(37.61) | 244(41.64) | 24(54.55) | 3(42.86) |  |
| Admission Type |  |  |  |  |  |  | <.001 |
| Outpatient | 4255(86.10) | 3665(87.51) | 91(77.78) | 454(77.47) | 39(88.64) | 6(85.71) |  |
| Emergency | 687(13.90) | 523(12.49) | 26(22.22) | 132(22.53) | 5(11.36) | 1(14.29) |  |
| Comorbidity Index | 0.00(0.00,14.00) | 0.00(0.00,14.00) | 4.00(0.00,9.00) | 0.00(0.00,9.00) | 7.50(2.25,18.50) | 0.00(0.00,2.00) | <.001 |
| Tumor Location |  |  |  |  |  |  | <.001 |
| Left-sided colon | 2415(48.87) | 2082(49.71) | 66(56.41) | 241(41.13) | 21(47.73) | 5(71.43) |  |
| Right-sided colon | 2151(43.52) | 1961(46.82) | 39(33.33) | 143(24.40) | 8(18.18) | 0(0.00) |  |
| Unspecified | 376(7.61) | 145(3.46) | 12(10.26) | 202(34.47) | 15(34.09) | 2(28.57) |  |
| Payment Method |  |  |  |  |  |  | <.001 |
| Self-pay | 1712(34.64) | 1692(40.40) | 19(16.24) | 0(0.00) | 1(2.27) | 0(0.00) |  |
| Medical Insurance | 3230(65.36) | 2496(59.60) | 98(83.76) | 586(100.00) | 43(97.73) | 7(100.00) |  |
| Surgical Approach |  |  |  |  |  |  | <.001 |
| Minimally Invasive | 3665(74.16) | 3159(75.43) | 76(64.96) | 389(66.38) | 37(84.09) | 4(57.14) |  |
| Open | 1277(25.84) | 1029(24.57) | 41(35.04) | 197(33.62) | 7(15.91) | 3(42.86) |  |
| Marital Status |  |  |  |  |  |  | 0.008 |
| Married | 4837(97.88) | 4112(98.19) | 113(96.58) | 563(96.08) | 42(95.45) | 7(100.00) |  |
| Single/Divorced/Widowed | 105(2.12) | 76(1.81) | 4(3.42) | 23(3.92) | 2(4.55) | 0(0.00) |  |
| Adverse Events |  |  |  |  |  |  | <.001 |
| YES | 129(2.61) | 73(1.74) | 13(11.11) | 40(6.83) | 2(4.55) | 1(14.29) |  |
| NO | 4813(97.39) | 4115(98.26) | 104(88.89) | 546(93.17) | 42(95.45) | 6(85.71) |  |

**Table S2 ICD-10 Codes and outcome fields for Identification of Major Postoperative Adverse Events**

| Complication | ICD-10 Codes |
| --- | --- |
| Acute Respiratory Failure | J96.0 |
|  | J96.9 |
| Acute Myocardial Infarction | I21 |
| Acute Renal injury | N17 |
| Acute Venous Embolism | I82 |
| Acute Infection | A04.7 |
|  | T80.2 |
| Sepsis | A22.7 |
|  | A26.7 |
|  | A40 |
|  | A41 |
|  | B37.7 |
| In-hospital Death | Patient's outcome |

**Figure S1. Point-score chart derived from the prediction model.**

Point-score chart derived from the full multivariable Firth regression model. Panel A shows the point assigned to each predictor, and Panel B shows the predicted risk corresponding to total points. Points were scaled so that the lowest-risk category within each predictor was assigned 0 points.
